# Supplementary material for: Nitrogen and phosphorus fertilisers optimise root morphology and soil nutrients in mixed annual grass and bean sown grassland in alpine regions
Source: PLoS One. 2025 Apr 15;20(4):e0321308. doi: 10.1371/journal.pone.0321308 (PMC11999165; doi:10.1371/journal.pone.0321308)
Supplement: Attachment — Submitted filename: response to reviewers.pdf [file pone.0321308.s002.pdf]

Dear editor,

Thanks for your letter and for reviewers' comments concerning our manuscript entitled "Influence of nitrogen and phosphorus additions on nutrient uptake in mixed annual grass and bean sown grassland in alpine region "(Manuscript ID: PONE-D-24-51070). Those co mments are allvaluable and helpful for revising and improving our paper. We havestudied all comments carefully and have made conscientious correction. The revised parts have be en marked in the paper.The responses to the reviewers' comments are as follows:

Comment 1.Please ensure that your manuscript meets PLOS ONE's style requirements, inc luding those for file naming. The PLOS ONE style templates can be found at.

Response: We really appreciate your efforts and comments on our manuscript. We have re viewed the formatting issues in the manuscript in light of the suggestions and have made changes with reference to the URL you provided.

Comment 2.We note that the grant information you provided in the 'Funding Information' and 'Financial Disclosure' sections do not match.

Response: We noticed that you mentioned a mismatch in some of the grant information, s o we've reworked it. Confirm that the grant information we provided is correct.

Comment 3.We note that your Data Availability Statement is currently as follows: [All re levant data are within the manuscript and its Supporting Information files

Response: Based on your suggestions, we have compiled the data in the article and will se nd it to you by email.

Comment 4.Although the overall aim of the paper is to investigate the multiple impacts o f nitrogen and phosphorus fertilization on mixed grasslands, the research question is p resented in a broad manner and lacks specificity. For example, does the study primari ly focus on improving forage nutrition, changes in soil nutrients, or optimizing root c haracteristics?

Response: We note that you have raised the issue of the broad and unfocussed formulation of your research question. After careful consideration, we have revised the title of the article to 'Nitrogen and phosphorus fertilisers optimise root morphology and soil nut rients in mixed annual grass and bean sown grassland in alpine regions' to refine th e research question. sown grassland in alpine regions' to refine the research question.

Nitrogen and phosphorus fertilisers optimise root morphology and soil nutrients in mixed annual grass and bean sown grassland in alpine regions

Comment 5.When discussing the mechanisms of nitrogen fertilization in increasing soil nu trients (e.g., total nitrogen and available nitrogen), the paper could incorporate the foll

owing reference to explore the contribution of microbial activity to nutrient accumulation: doi: 10.1016/j.apsoil.2024.105498.

Response: We really appreciate your efforts and comments on our manuscript. Thinking that the comments you gave had a very important positive effect on the article, we listened to you and added the literature you recommended.

7 Gang F, Yong H. Responses of soil fungal and bacterial communities to long-term organic and inorganic nitrogenous fertilizers in an alpine agriculture. Applied Soil Ecology 2024,7(1): 105498. https://doi.org/10.1016/j.apsoil.2024.105498

Comment 6. In the background and discussion of soil nutrient responses, the study could be linked with this meta-analysis to analyze the general patterns of nitrogen addition on alpine grasslands and highlight how regional differences or fertilization quantities may have contributed to the specific results:Fu Gang, Shen Zhenxi. DOI: 10.1016/j.apsoil.2017.02.016.

Response: We really appreciate your efforts and comments on our manuscript. Thinking that the comments you gave had a very important positive effect on the article, we listened to you and added the literature you recommended.

32 Bengjamin A. Mc G. Maxim J. Schlossgerg. Fine-scale spatial analysis of soil moisture and entomopathogenic nematode distribution following release in wetting agent-treated turf. Applied Soil Ecology.2017,6(1):52-61. https://doi.org/10.1016/j.apsoil.2017.02.016.

Comment 7.When discussing plant responses to nitrogen fertilization in relation to the special climate conditions of the study area, the paper could refer to the following study to support general conclusions about plant responses in alpine regions: doi:10.1007/s00344-016-9595-0.

Response: We really appreciate your efforts and comments on our manuscript. Thinking that the comments you gave had a very important positive effect on the article, we listened to you and added the literature you recommended.

35 Gang F. Zhen X S. Response of Alpine Plants to Nitrogen Addition on the Tibetan Plateau: A Meta-analysis.Journal of Plant Growth Regulation. 2016,3(35):974-979. https://doi: 10.1007/s00344-016-9595-0.

Comment 8.The sentence “The addition of nitrogen and phosphorus nutrients did not significantly advantage the enhancement of forage nutrients in grass-bean mixes compared to no fertilization, without fertilization could be more beneficial to the growth of grass-bean mixes” is overly complex and unclear. A clearer version might be: "The addition of nitrogen and phosphorus did not significantly enhance forage nutrients in grass-legume mixes compared to no fertilization, and no fertilization appeared more beneficial for the growth of grass-legume mixes."

Response: We really appreciate your efforts and comments on our manuscript. Based on your suggestions, we have made the appropriate changes in the article.

Abstract: The grass-bean hybrid system in alpine regions was an important method to increase the root system and nutrients of grasses, however, there was no clear conclusion from the research on whether the nitrogen——phosphorus fertiliser additions in alpine regions had an enhancement effect on the root system and nutrients of the grass-bean hybrids.Through the establishment of an artificial grass-bean mix in an alpine region at 4,270 meters above sea level, we examined the effects of exogenous fertilizer additions on forage roots and nutrients in an annual grass-legume forage mix system using (fertilizer application: $A_{1\sim4}$ vs. mix ratio $B_{1\sim5}$).The results illustrate that: the addition of nitrogen and phosphorus did not significantly increase forage nutrients in grass-bean mixtures compared to no fertiliser, which appeared to be more favourable; nitrogen——phosphorus nutrients increased the root length and root projection area of forage, and also increased the nutrient content of the soil.The forage CP, TN, TC, and TP contents as well as the soil nutrient contents gradually increased as the fraction of legume forage increased under various mixing ratios. However, as the percentage of legumes increased, root morphology trended downward, and mixed treatments outperformed the monoculture control.The study's findings were as follows: In the nitrogen——phosphorus fertilizer addition test of annual grass and bean mixed sowing in alpine areas, nitrogen can increase the surface area and projected area of the root system, while phosphorus can increase the length of the root system and have significant improvement effects on soil nutrients. However, there is no clear benefit in terms of improving the forage's nutrient content. This could offer direction for choosing fertilizers and improving soil fertility in grasslands that are combined with annual grass and beans.

Comment 9.'In the phrase "nitrogen-phosphorus mixing," a long dash should be used for clarity and formality: "nitrogen—phosphorus mixing."

Response: We really appreciate your efforts and comments on our manuscript. Based on your suggestions, we have made the appropriate changes in the article.

Comment 10.The citation format for references should be consistent. Some references include URLs while others do not.

Response: Thank you very much for your efforts and comments on our manuscript. We have rechecked the references and reworked them according to the editorial formatting requirements.

Comment 11"The effect of phosphorus was studied by applying phosphorus fertilizer to the plots" could be rephrased as: "The effect of phosphorus was studied through the application of phosphorus fertilizer."

Response: We really appreciate your efforts and comments on our manuscript. Based on your suggestions, we have made the appropriate changes in the article.

Comment 12 "Based on the experiment, it is concluded that..." can be simplified to: "The experiment concluded that..."

Response: We really appreciate your efforts and comments on our manuscript. Based on your suggestions, we have made the appropriate changes in the article.

Comment 13 The figure captions should not only label the figures but also attempt to explain the overall content and findings of the images.

Response: We really appreciate your efforts and comments on our manuscript. Based on your suggestions, we have made the appropriate changes in the images of the article. For example:

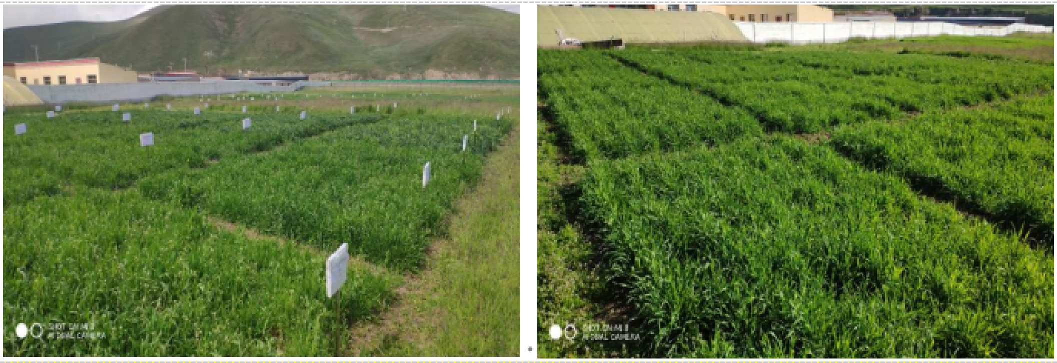

Fig. 1 Layout of oats and forage peas grown in this experimental site (elevation 4,270m, 15m^2 per treatment)

Table 2 Two-factor ANOVA for forage quality by fertilisation and mix ratio

Source of variation	F-value						
	NDF	ADF	CP	SS	TN	TC	TP
Fertilise	16.82**	22.64**	91.29**	361.49**	92.60**	12.51**	54.18**
Mixed sowing ratio	88.67**	73.91**	272.34**	331.16**	185.90**	6.89**	35.47**
Fertiliser × Mixed sowing ratio	25.40**	44.72**	60.05**	238.90**	91.35**	25.01**	61.46**

Note: * indicates significant difference (P<*0.05*), ** [illegible]es highly significant difference (P<*0.01*).NDF=Neutral Detergent Fibres，ADF=Acid Detergent Fibres，CP=Crude Protein，SS=Soluble Sugar，TN=Total Nitrogen，TC=Total Carbon，TP=Total Phosphorus.

[illegible]
